# High fibrinogen levels are associated with poor survival in patients with liposarcoma

**DOI:** 10.1038/s41598-023-31527-6

**Published:** 2023-05-27

**Authors:** L. S. Peschek, Gerhard M. Hobusch, P. T. Funovics, M. Willegger, M. P. Schmid, G. Amann, W. Lamm, Th. Brodowicz, C. Ay, R. Windhager, J. Panotopoulos

**Affiliations:** 1 Department of Orthopaedics, Medical University of Vienna, Währinger Gürtel 18-20, 1090 Vienna, Austria; 2 Department of Radiation Oncology, Medical University of Vienna, Vienna, Austria; 3 Clinical Institute of Pathology, Medical University of Vienna, Vienna, Austria; 4 Department of Medicine I, Clinical Division of Oncology, Medical University of Vienna, Vienna, Austria; 5 Department of Medicine I, Clinical Division of Hematology and Hemostaseology, Medical University of Vienna, Vienna, Austria

**Keywords:** Sarcoma, Prognostic markers

## Abstract

The aim of this study was to evaluate whether (preoperative) plasma levels of fibrinogen, an essential clotting and acute phase protein, are associated with the prognosis of patients with a liposarcoma, a subtype of sarcoma derived from adipose tissue. We performed a retrospective cohort study of 158 patients with liposarcoma treated at the Department of Orthopaedics of the Medical University of Vienna in Austria from May 1994 to October 2021. Kaplan–Meier curves as well as uni- and multivariable Cox proportional hazard models were performed to evaluate the association between fibrinogen levels and overall survival. Elevated fibrinogen was associated with adverse overall survival in cause specific hazards analysis of mortality (hazard ratio [HR] per 10 mg/dL increase: 1.04; 95% CI 1.02–1.06; p < 0.001). This association prevailed in multivariable analysis after adjustment for AJCC tumor stage (HR 1.03; 95% CI 1.01–1.05; p = 0.013). Increasing levels of fibrinogen, a routinely available and inexpensive parameter, predicts the risk of mortality in patients with liposarcoma.

## Introduction

Establishing survival-prognosis of the individual patient is of uppermost interest, so there is intense focus on finding tumor-related prognostic factors to influence clinical decisions on operative treatment and the further therapeutic concept^1–3^.

Liposarcomas represent a spectrum of malignant tumors with adipocytic differentiation and are one of the most common subtypes of soft tissue sarcoma (STS). The 5-year survival rates are between 57 and 95%. The clinical presentation is versatile, appearing on all body parts, but cumulatively on extremities and the retroperitoneum and the course of the disease is difficult to predict^4–7^.

The main histological subtypes are: atypical lipomatous tumor (ALT)/well differentiated liposarcoma, myxoid/round cell liposarcoma, dedifferentiated liposarcoma and pleomorphic liposarcoma^4,8^. The pathological subclassification and histologic grade are key prognostic factors for survival (Hannibal, Rutkowski): while ALT only carry a risk of local recurrence, the pleomorphic and dedifferentiated liposarcomas are high-grade malignancies with a substantial risk of metastatic disease^4,7,9^. Tumor size, depth, site, grade, age at diagnosis and resection margins have been associated with overall survival (OS) in STS^6,7^.

In recent studies the concept of the involvement of systemic inflammation and acute phase proteins in cancer progression and metastasis has been postulated. Specifically, elevated preoperative CRP and neutrophil/lymphocyte ratio (NLR) as markers of systemic inflammatory response have been found to be associated with decreased overall survival in various cancers^10–18^.

Furthermore, lower levels of serum albumin are considered to be an indicator of current systemic immune response to tumor cell products and inflammatory cytokines. Correspondingly, biomarkers of kidney dysfunction were identified to predict inpatient mortality. Elevated serum creatinine, low albumin, and a decreased albumin–creatinine ratio (ACR) were found to be negative prognostic factor with worse disease specific survival in patients with myofibroblastic and fibroblastic sarcoma as well as liposarcoma^19,20^.

Interestingly, also a link between certain proteins of haemostasis and tumor progression was evidenced in previous studies^17,21–23^. Ay et al. showed that high d-dimer levels, as a biomarker indicating the activation of haemostasis and fibrinolysis, are associated with poor overall survival and increased mortality risk in cancer patients^24,25^.

In observational studies, fibrinogen, which is an essential protein for blood clot formation and also an acute phase protein, was described as an useful prognostic biomarker for several malignancies^17,21,26,27^.

The impact of plasma levels of fibrinogen on risk of mortality and survival in patients with soft tissue sarcoma has been described, however, the value in association with liposarcoma has not yet been elucidated.

The aim of the present study was to investigate plasma levels of fibrinogen as a prognostic biomarker in patients with liposarcoma.

## Patients and methods

The study population consisted of a total of 184 patients with histologically confirmed liposarcoma, that were treated at the Department of Orthopaedic Surgery, Medical University of Vienna from 1994 to 2021. All patients were followed-up until July 2021 at our department with a standardized interval, which contains examinations every 3 months during the first 3 years, every 6 months in year 4 and 5 and in 12-month intervals after that.

Post-operative surveillance incorporated clinical examination, ultrasound of the abdomen, computed tomography scans of the thorax and local magnetic resonance imaging.

A cut-off of 12 weeks was defined and excluded all patients (patients who have been operated on account of their sarcoma at an outside clinic prior referral to our department because of insufficient excision or nonradical resection margins) with a longer duration between index surgery and referral to our clinic. The study population incorporates 158 patients with complete clinical pathological data (i.e. the final study population) and retrospectively, the corresponding data was collected from medical reports (sex, age, laboratory parameters, tumor site, tumor size, tumor depth, histology, resection margins, tumor stage, neoadjuvant and adjuvant radiotherapy, adjuvant chemotherapy, local recurrence).

In the study population, 11 patients (6.96%) received preoperative radiotherapy.

Laboratory data from routine inquiries were acquired within 2 weeks before surgical treatment and the minimum interval to radiotherapy is 22 days.

Histopathological analysation, as well as diagnoses, were done in accordance with to the current WHO classification for soft tissue and bone tumors and validated at our clinic by an proficient pathologist specialised in STS^8^. According to AJCC criteria, tumor stage was generated^29^, Resection margins were allocated following Enneking et al.^28^ and grading (i.e. G1–G3) according to the French Federation of Cancer Centres Sarcoma Group (FNCLCC) grading system)^30^.

This work was given approval by our local ethics committee of the Medical University of Vienna, Austria. We conform that all methods took place in accordance with relevant guidelines and regulations, under informed consent from all participants.

Level of evidence III (retrospective cohort study).

### Statistical analysis

For any statistical analyses and graphical visualization IBM SPSS Statistics, Version 27, SPSS Inc, Chicago, IL, USA, was utilized. Continuous data was summarized, using mean values, medians and ranges and categorical data by absolute frequencies and percentages. For the calculation of correlations, Spearmann’s correlation coefficient was applied. Baseline was determined as the day of first diagnosis and the endpoint (overall survival) was defined as death from any cause, which in this study could carefully be considered as equal of death of disease. We considered follow-up time as the timespan from index surgery to death or last known alive. Calculation of survival probabilities were computed with the Kaplan–Meier product limit estimator. For this purpose, serum Fibrinogen levels were categorized into < 450 and ≥ 450 mg/dL. Comparison of the survival functions of two or more patient groups, were applied with the log-rank test. Further, for the evaluation of the relation between baseline variables and survival, uni- and multivariable Cox proportional hazards regression models were fitted. Calculation of the multivariable model was computed with the co-variable AJCC tumor stage. P values < 0.05 were considered to indicate statistical significance.

## Results

The median age of the total study population was 66.4 (range 7–99) years and the median follow-up time 37.5 months (range 1–228.8 months). The median baseline fibrinogen level (g/L) was 353.0 (range 132.0–956.0).

The primary tumor was located as follows: extremities in 137 cases (86.7%), trunk in 12 (7.7%) and other location in 9 (5.7%).

The baseline study population characteristics are shown in Table 1.

**Table 1:** Baseline characteristics of the study population.

	Number (n)	%
Sex		
Male	90	57
Female	68	43
Tumor site		
Extremities	137	86.7
Trunk	12	7.6
Other	9	5.7
Depth		
Epifascial	19	12.0
Subfascial	132	83.5
Not known	7	4.4
Histology		
Highly differentiated	44	27.8
Myxoid	77	48.7
Dedifferentiated	11	7.0
Pleomorph	13	8.2
NOS	13	8.2
Grading		
G1	55	34.8
G2	60	38.0
G3	43	27.2
Resection margins		
Wide	61	38.6
Focal marginal	43	27.2
Marginal	27	17.1
Focal intralesional	15	9.5
Intralesional	5	3.2
Not known	7	4.4
Local recurrence	14	8.9
Metastasis	18	11. 4
Tumor stage (AJCC)		
Stage IA	2	2.5
Stage IB	24	1.3
Stage II	6	15.2
Stage IIIA	31	19.6
Stage IIIB	89	56.3
Stage IV	2	1.3
Not known	4	2.5
Tumor size		
≤ 2 cm	1	0.6
> 2 ≤ 5 cm	5	3.1
> 5 cm	134	84.8
Not known	18	11.4
Adjuvant therapy		
None	44	27.8
Chemotherapy only	2	1.3
Radiation only	80	50.6
Both	26	16.5
Neoadjuvant radiation	11	6.69
Not known	6	3.8
Age at baseline (years)	Mean	Range
	66.4	7–99
Laboratory parameters	Median	Range
Fibrinogen (mg/dL)	353.0	132.0–956.0
C-reactive protein (mg/dL)	1.16	0.03–21.0
Haemoglobin (g/dL)	10.1	3.0–21.2
Alkalic phosphatase (U/L)	72.5	32.0–238.0
NLR	7.4	0.1–27.8

In cause specific hazards analysis of overall survival, elevated fibrinogen was associated with adverse overall survival (hazard ratio [HR] per 10 mg/dL increase: 1.04; 95% CI 1.02–1.06; p < 0.001). This association prevailed after adjustment for AJCC tumor stage (HR 1.03; 95% CI 1.01–1.05; p = 0.013) in multivariable analysis.

Fibrinogen is highly correlated with both CRP, NLR and Alkalic Phosphatase (ρ = 0.38; p < 0.001, ρ = 0.18; p = 0.024 and ρ = 0.39; p < 0.001). Moreover, there is strong evidence for an inverse correlation between haemoglobin and fibrinogen (ρ = − 0.19, p = 0.014). There was no correlation with tumor size.

**Figure 1:**
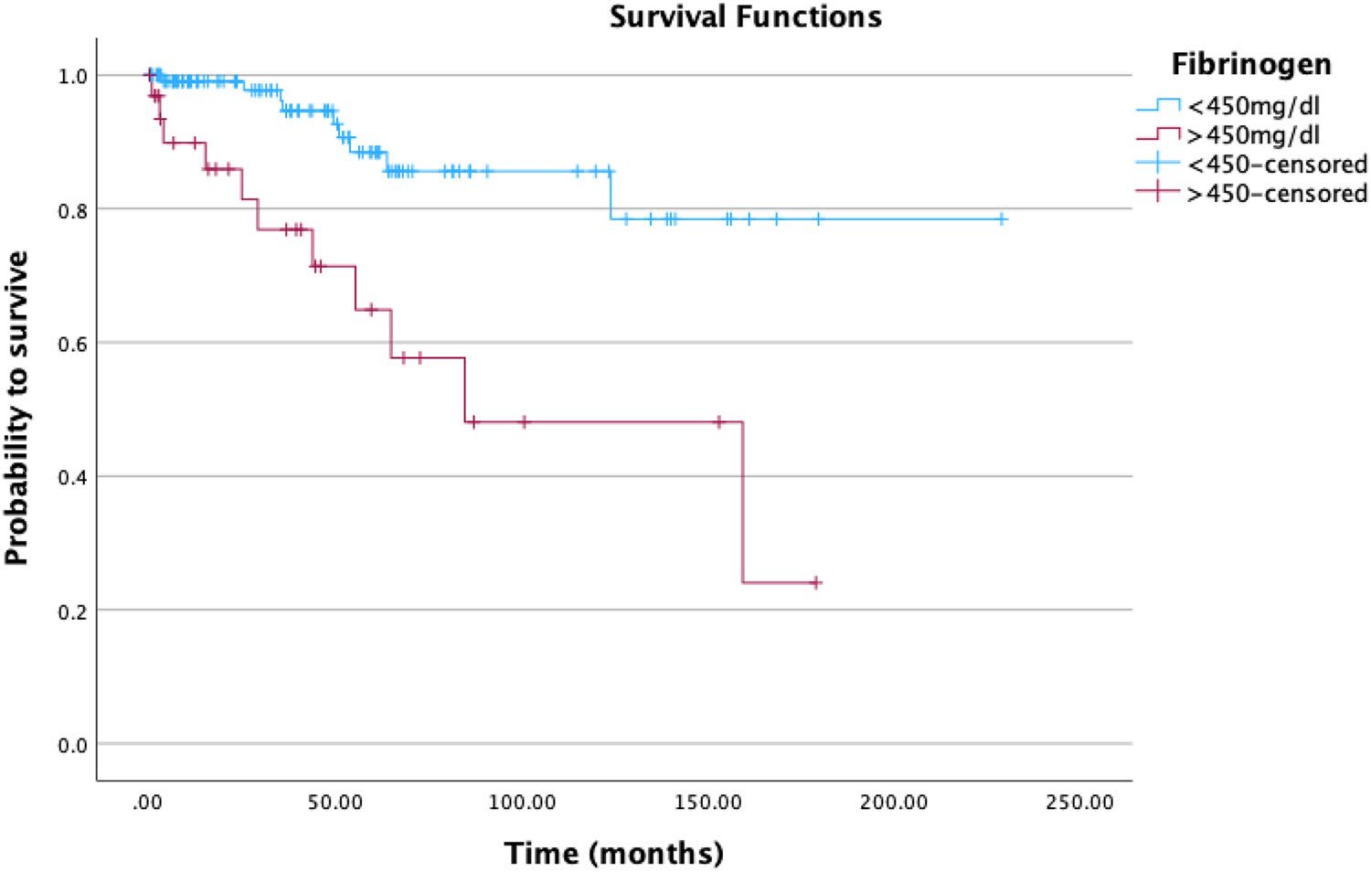
Kaplan–Meier survival analysis in patients with liposarcoma distributed by preoperative fibrinogen levels.

Patients with pre-operative fibrinogen levels ≥ 450 mg/dL (n = 33) had a lower survival rate than patients with fibrinogen levels < 450 mg/dL (n = 125) (Log Rank p < 0.001) (survival rates of 78.5% vs. 24.0%). Kaplan–Meier survival analysis is shown in Fig. 1. The overall survival rate was 77.9%.

## Discussion

In the present study, we identified a significant association between plasma fibrinogen and overall survival.

Fibrinogen is a key protein in the coagulation pathway and represents one of the major acute phase proteins^31^. Moreover, plasma fibrinogen has been reported to be synthesized and overexpressed in human neoplasia cells^32^. Fibrinogen itself induces the synthesis of pro-inflammatory cytokines and modulates immune activity^31,33^. Cancer cells may interact directly and indirectly with host inflammatory cells^33^. In recent studies, the concept of the involvement of systemic inflammation in cancer progression and metastasis has been postulated. Specifically, elevated preoperative CRP as marker of systemic inflammatory response have been found to be associated with decreased overall survival^12,16,34–40^.

Previous reports showed that elevated fibrinogen levels were associated with adverse outcome for the following reasons: Fibrinogen may enhance tumor progression and the development of tumor spread through the following mechanism: tumor cells prefer to adhere to fibrinogen and secondly fibrinogen enhances the adhesion of tumor cells to platelets which may protect tumor cells from immune answer^41–43^.

Plasma fibrinogen was described as useful prognostic biomarker for other malignancies (Ovarian Cancer^44^, Gastric cancer^45^ Renal cell Carcinoma^21^, Hepatocellular Carcinoma^46^, solid tumors^27^ and also for Soft tissue Sarcoma in general^17^ but not for Liposarcoma per se. Our findings are in line with the studies mentioned before and are clinically plausible.

While we are aware of the limitations of the present study (retrospective design, sample size prevalent cases), the strength of our report is that we report about a single center population of liposarcoma patients, although histological subtypes of liposarcoma have to be distinguished.

Furthermore, a possible effect of preoperative radiotherapy on fibrinogen levels needs to be addressed. In fact, there is some data on the increase of fibrinogen synthesis after irradiation therapy. Maximum increase is reached about 4–6 days after irradiation^47^.

Since the half-life of fibrinogen is about 3–5 days, the minimum interval between irradiation and blood sampling was at least 22 days and only a few patients have received neoadjuvant irradiation, our results may be assumed to be valid^48^.

Furthermore, there is a minimum interval of 5 weeks between last irradiation and performed surgery.

Previous studies reported that there is no significant difference in outcome when re-resection is performed within 12 weeks after initial surgery^49^. The inclusion of the prevalent cases in the present study may not reflect a survivorship bias because we set the cut off of the prevalent cases of 12 weeks and excluded all patients that had a longer duration between initial surgery and re-resection.

## Conclusion

Fibrinogen is easy to assess and is an established laboratory parameter used in daily clinical routine. A benefit of this biomarker could be to create an individual risk profile and predict the clinical outcome and in consequence have an influence on the extent of treatment and posttreatment morbidity.

## Data Availability

The data was anonymized and stored according to the guidelines of the Medical University of Vienna. The datasets analysed during the current study are available from the corresponding author on reasonable request.
